# 680 Utilizing a Quality Audit Tool to Mitigate Over-Resuscitation Incidence

**DOI:** 10.1093/jbcr/iraf019.309

**Published:** 2025-04-01

**Authors:** Lindsay Desantis, Laurin Proctor, Samantha Allbritton, Lily Daniali, Ryan Endress, Wojciech Przylecki, Benson Pulikkottil

**Affiliations:** HCA HealthONE Swedish; HCA HealthONE Swedish; HCA HealthONE Swedish; HCA HealthONE Swedish; HCA HealthONE Swedish; HCA HealthONE Swedish; HCA HealthONE Swedish

## Abstract

**Introduction:**

Data analysis during the acute care phase of fluid resuscitation for severely burned patients can mitigate complications associated with over-resuscitation and reduce mortality rates. The development and implementation of an audit tool by a burn quality team facilitated data analysis and identification of key factors influencing over-resuscitation in this critically injured population.

**Methods:**

At the studied institution, fluid resuscitation is administered to patients with greater than or equal to 20% total body surface area burned (TBSA). Over-resuscitation is defined as a fluid administration rate of greater than or equal to 5 ml per kg per TBSA per hour during the resuscitation phase, accompanied by associated complications. A quality audit tool was developed and applied to all fluid resuscitations over a three-year period to identify contributing factors to over-resuscitation. The tool allowed for hour-by-hour analysis of patient data, including vital signs, hemodynamic measurements, urine output, laboratory values, resuscitation rates, and cumulative fluid intake. Each hour during the first 24 hours post-injury was analyzed using this tool, enabling the quality team to identify trends in pre-hospital treatment interventions and inpatient resuscitation that predisposed patients to negative outcomes, as well as to implement strategies to mitigate these outcomes.

**Results:**

Within the defined population, the incidence of over-resuscitation decreased by 62.5% within one year post-implementation and was eliminated entirely within three years. Additionally, the incidence of abdominal compartment syndrome was zero, and the mortality rate of resuscitated patients decreased by 34% over the same three-year period. Key opportunities identified in this study included streamlined inter-departmental communication, focused educational initiatives, improved staff retention, and the addition of a dedicated burn educator to the burn center.

**Conclusions:**

The implementation of a quality audit tool to evaluate fluid resuscitation can effectively decrease the incidence of over-resuscitation in burn centers. Continued efforts in data analysis may further identify essential treatment interventions for critically injured burn patients.

**Applicability of Research to Practice:**

Focused burn quality department efforts in data capture and analysis can lead to the identification of key opportunities in clinical intervention and targeted educational initiatives for clinicians.

**Funding for the Study:**

N/A

